# Efficacy and safety of prophylactic intravenous calcium supplementation in patients with secondary hyperparathyroidism after parathyroidectomy: a retrospective study

**DOI:** 10.3389/fendo.2025.1652418

**Published:** 2025-11-24

**Authors:** Yao Pan, Jiaqi Zhu, Zhenglong Wu, Dengfeng Wu

**Affiliations:** Department of Thyroid Surgery, The Affiliated Wuxi People's Hospital of Nanjing Medical University, Wuxi People's Hospital, Wuxi Medical Center, Nanjing Medical University, Wuxi, Jiangsu, China

**Keywords:** severe hypocalcemia, secondary hyperparathyroidism, parathyroidectomy, alkaline phosphatase, dialysis

## Abstract

**Objective:**

Severe hypocalcemia is a common complication in patients with secondary hyperparathyroidism (SHPT) after parathyroidectomy (PTX). The necessity of postoperative prophylactic intravenous calcium supplementation and the corresponding strategies are controversial. This study aimed to analyze the efficacy and safety of prophylactic intravenous calcium supplementation after PTX.

**Methods:**

We retrospectively reviewed the clinical data of 125 patients with SHPT who underwent PTX between January 2020 and December 2023. Patients were divided into prophylactic intravenous calcium supplementation group (group A, 94 cases) and non-prophylactic intravenous calcium supplementation (group B, 31 cases). The baseline characteristics, laboratory data, surgical outcomes of the two groups were compared. Binary logistic regression analysis was used to identify risk factors for severe hypocalcemia and hypercalcemia after calcium supplementation.

**Results:**

The incidence of severe hypocalcemia in group A (30 cases, 31.9%) was lower than that in group B (19 cases, 61.3%) (P = 0.004). Binary logistic regression analysis revealed that non-prophylactic intravenous calcium supplementation, high level of preoperative parathyroid hormone (PTH) and alkaline phosphatase (ALP), and low level of preoperative serum calcium were risk factors for severe hypocalcemia. Preoperative high level of ALP was a negative predictor for hypercalcemia after calcium supplementation.

**Conclusions:**

Postoperative prophylactic intravenous calcium supplementation, followed by dynamic dosage adjustments based on serum calcium levels, in addition to routine oral calcium and calcitriol supplementation, could significantly reduce the incidence of severe hypocalcemia without substantially increasing the risk of hypercalcemia.

## 1 Introduction

Secondary hyperparathyroidism (SHPT) is a common complication of chronic kidney disease (CKD). It is characterized by significantly elevated levels of serum parathyroid hormone (PTH) (1). In end-stage kidney disease (ESKD), PTH secretion is stimulated by decreased levels of 1,25 (OH)_2_D values, and increased levels of serum phosphorus and fibroblast growth factor 23 (FGF-23) values, contributing to the release of calcium and phosphorus in bone. This causes high turnover renal osteodystrophy, which manifests as osteoporosis and even pathological fractures. Additionally, vascular calcification and the increase of phosphate and FGF23 levels promote the occurrence of cardiovascular disease (2, 3). Studies have shown that serum PTH level is an independent risk factor for fracture, cardiovascular events and all-cause mortality in CKD patients (4).

Medical therapies for SHPT includes calcium agents, non-calcium-based phosphate binders, vitamin D and analogs, and calcimimetics agents. They help patients maintain appropriate serum calcium and phosphate levels in order to suppress the secretion of PTH and improve clinical symptoms (1). Although pharmaceutical therapies could largely delay SHPT progression, there are still a considerable number of patients who have failed pharmaceutic therapy because of drug resistance, inability to tolerate the side effects (e.g., gastrointestinal reactions brought by cinacalcet) and other reasons (5, 6). Surgical interventions should be considered for them.

Parathyroidectomy (PTX), regardless of the surgical technique, has been proved to significantly improve the quality of life and survival for SHPT patients who have failed pharmaceutic therapy (7, 8). Hypocalcemia is a common complication of PTX, caused by the rapid decline in PTH levels after surgery, which leads to shift of calcium from the bloodstream into bones (6). Hypocalcemia after PTX is also known as hungry bone syndrome (HBS), which is defined as serum calcium drops to < 2.1mmol/L and/or hypocalcemia lasting for more than 4 days after surgery (9). In severe hypocalcemia, potential life-threatening conditions such as bronchospasm and arrhythmia may occur (7, 10). Therefore, prediction and early treatment of severe hypocalcemia are essential. However, there is considerable variability in the postoperative monitoring of serum calcium levels, whether to provide prophylactic intravenous calcium supplementation after PTX, and the corresponding strategies among current studies, closely related to the experiences and practical conditions of different centers. Some scholars also express concerns about the safety of intravenous calcium supplementation Therefore, we carried out this retrospective study with the aim of analyzing the efficacy and safety of postoperative prophylactic intravenous calcium supplementation and identifying the high-risk populations for severe hypercalcemia.

## 2 Materials and methods

### 2.1 Research subjects

After the approval of the Review Board of Wuxi People’s Hospital of Nanjing Medical University (Approval Number: HG-2024-132, Approval Date: August 15th, 2024), we included patients who underwent PTX for SHPT at The Affiliated between January 2020 and December 2023. The research was performed in accordance with relevant guidelines and regulations. All patients or their relatives signed informed consent forms to participate in the study. Research involving human research participants have been performed in accordance with the Declaration of Helsinki. The exclusion criteria were incomplete PTX and severe lack of biochemical data. We collected data on patient demographics, including gender, age and dialysis protocol, preoperative long-term medications for mineral and bone disorder, and perioperative biochemical values such as PTH, serum calcium corrected for albumin concentration, serum phosphorus and alkaline phosphatase (ALP), and surgical information, including number of removed parathyroid glands and pathological results. We also focused on the details of postoperative calcium supplementation, hypocalcemia and its severity, and hypercalcemia after calcium supplementation.

### 2.2 Preoperative preparation

The indications for PTX were continuous elevated PTH >800pg/ml for more than 6 months with ineffective medications, or obvious symptoms including arthralgia, itching, and asthenia. Each patient took 99m-technetiumsestamibi scintigraphy combined with computed tomography (SPECT/CT) and ultrasound examination to locate parathyroid glands and investigate any possible ectopic parathyroid glands.

### 2.3 Surgical procedure

**Figure 1:**
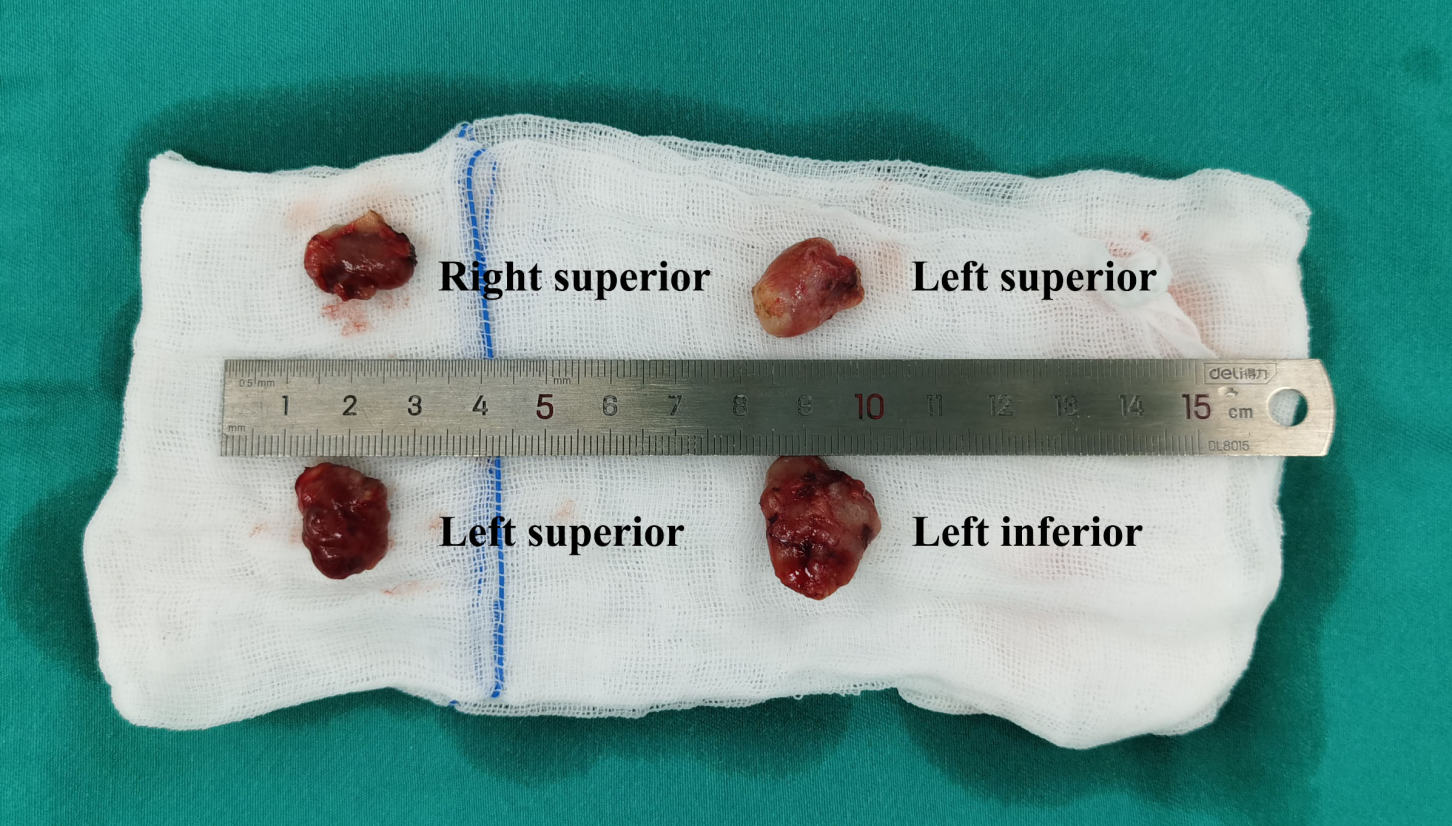
Four parathyroid glands removed in parathyroidectomy. The pathological result of them was diffuse hyperplasia.

During the surgery, we first searched for and removed all parathyroid glands (**Figure 1**). If four or more parathyroid glands were found, autotransplantation would be performed in order to prevent permanent hypothyroidism. One parathyroid gland that was closest to normal was selected, sliced into 8~12 pieces of homogenates with 1mm^3^ size and implanted in brachioradialis muscle of the non-arteriovenous fistula side. If less than 4 parathyroid glands were found, all identified parathyroid glands were removed without autotransplantation. Frozen pathological examination and intraoperative PTH monitoring were routinely used. Intraoperative PTH were measured at 10 minutes after the removal of the last parathyroid gland. An at least 60% drop compared to preoperative level was considered as cure for SHPT.

### 2.4 Perioperative management

If the patients had not received any form of active vitamin D analogs prior to surgery, they were prescribed 1 mcg of calcitriol twice a day for 2 days before PTX. Patients on hemodialysis underwent dialysis using a dialysate containing 1.5 mmol/L ionized calcium on the day before and after PTX, and thereafter, three times a week. Patients on peritoneal dialysis continued on dialysis after surgery using a peritoneal dialysis solution with 1.25 mmol/L ionized calcium. Central venous catheters (CVC) were inserted to facilitate calcium supplementation.

**Figure 2:**
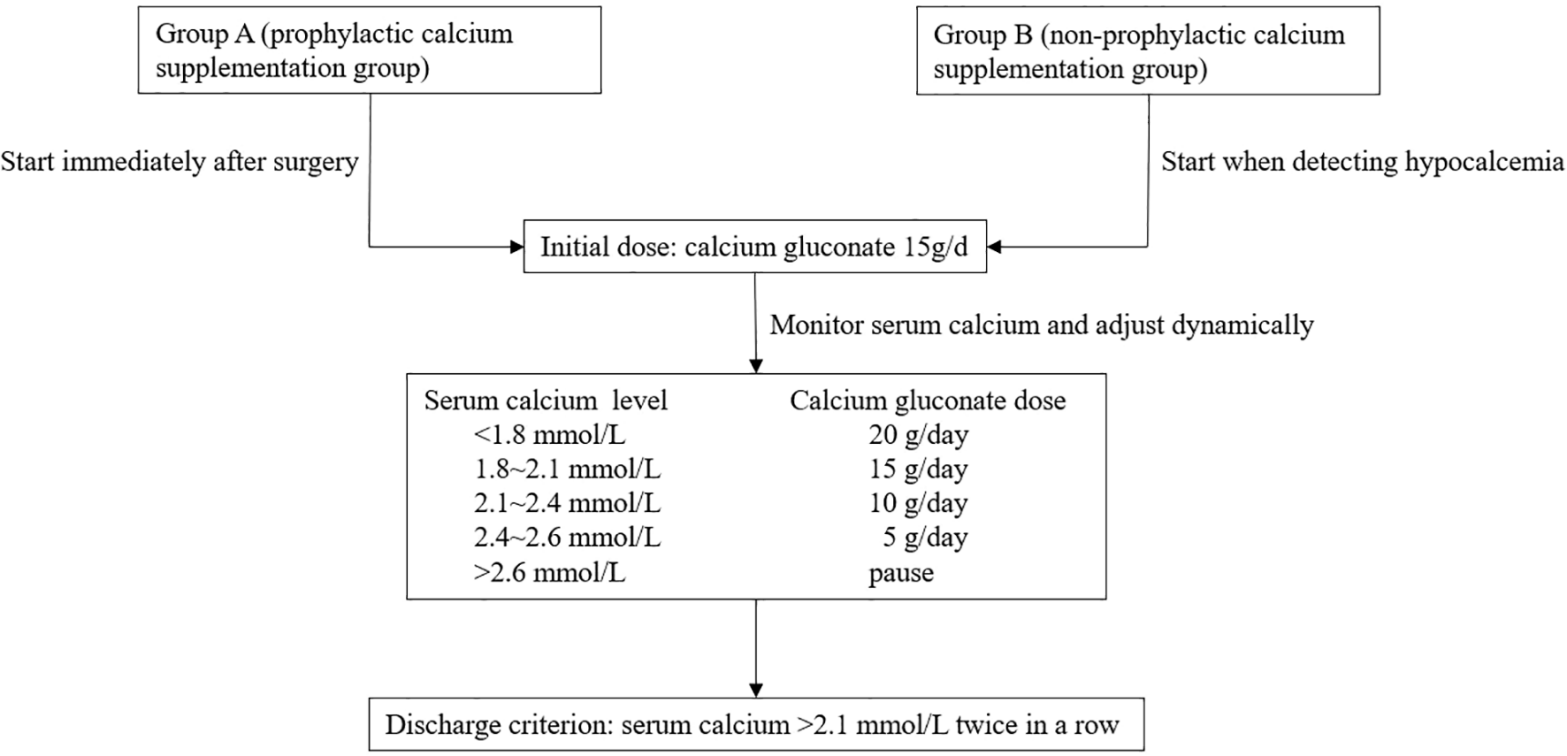
Flowchart of intravenous calcium supplementation for patients undergoing parathyroidectomy.

Serum PTH and electrolyte levels were monitored at 4 hours postoperatively, and on the morning of postoperative day 1 and day 3. These indications were additionally monitored every two days if serum calcium was > 2.1 mmol/L and without the presence of symptoms of hypocalcemia, such as tingling in the extremities and muscle cramps, otherwise these measurements were taken daily. Severe hypocalcemia was defined as serum calcium < 1.8 mmol/L. After surgery, all patients were given 1 mcg of calcitriol twice per day and 1.5 g of calcium carbonate (600 mg of elemental calcium) three times per day once oral medication was feasible, unless hypercalcemia was detected. Medication dosages were adjusted based on the clinical symptoms and calcium levels, with the goal of maintaining a calcium level between 2.10 and 2.60 mmol/L. There were two methods for postoperative intravenous calcium supplementation, depending on the habits of different physicians. The first was to start intravenous calcium supplement immediately after surgery, and the second was to start when detecting hypocalcemia. According to the start timing of postoperative calcium supplementation, patients were divided into prophylactic intravenous calcium supplementation group (group A) and non-prophylactic intravenous calcium supplementation (group B). **Figure 2** presented the protocol used in our center for intravenous calcium supplementation. Intravenous calcium supplementation was administered via CVC using 10% calcium gluconate, which would be diluted 1:1 with normal saline. The initial dosage was standardized at 15 g/day, with adjustments guided by serum calcium levels, specifically: 20 g/day for serum calcium <1.8 mmol/L, 15 g/day for 1.8~2.1 mmol/L, 10 g/day for 2.1~2.4 mmol/L, and 5 g/day for 2.4~2.6 mmol/L. Infusion was temporarily discontinued when serum calcium exceeded 2.6 mmol/L. Intravenous calcium supplementation usually last at least 3 days, unless hypercalcemia (serum calcium >2.6 mmol/L) happened. The discharge criterion was monitored serum calcium >2.1 mmol/L twice in a row.

### 2.5 Statistical analysis

SPSS 23.0 was used for statistical analysis. Continuous data were expressed as mean and standard deviation when normal distributed or as median and quartile. Student’s t-test was used compare the differences of variables between two groups for quantitative variables, while chi-squared, fisher’s exact test or Mann-Whitney U test were used for quantitative variables, where appropriate. Binary logistic regression analysis was used to identify risk factors for postoperative severe hypocalcemia and hypercalcemia after calcium supplementation, and were reported as odds ratio (OR) and 95% confidence index (95% CI). All baseline characteristics, preoperative laboratory data and surgical data were included for univariate regression analysis. Then stepwise backward multivariate regression analysis was performed on variables with statistical differences. All statistical tests were two-sided. It was considered statistically significant when P <0.05.

## 3 Results

A total of 125 patients who met the inclusion criteria were included in this study (1 case of recurrent SHPT, 2 cases with incomplete data and 1 case with incomplete parathyroidectomy were excluded). Ninety-four patients were divided into Group A (prophylactic intravenous calcium supplementation group) and thirty-one into Group B (non-prophylactic intravenous calcium supplementation group). The baseline characteristics, preoperative laboratory data and surgical data, and the preoperative long-term medications for mineral and bone disorder were presented in **Tables 1** and **2**, respectively. There were no significant differences in gender, age, cause of kidney disease, dialysis modality and duration, preoperative biochemistry, pathological results and preoperative medications between the two groups (P >0.05). Except for 2 cases (2.1%) in Group A and 2 cases (6.5%) in Group B who underwent TPTX without autotransplantation due to only 3 parathyroid glands were detected, all other patients underwent TPTX+AT.

**Table 1:** Baseline characteristics, preoperative laboratory data and surgical data of patient undergoing parathyroidectomy, stratified by the type of postoperative calcium supplementation.

Variable	Group A (prophylactic intravenous calcium supplementation group) (n=94)	Group B (non-prophylactic intravenous calcium supplementation group) (n=31)	P value
Female gender, n (%)	35 (37.2)	16 (51.6)	0.158
Age, year, mean (SD)	52.0 (11.7)	50.2 (10.4)	0.454
End-stage kidney disease cause, n (%)			0.106
Glomerulonephritis	37 (39.4)	8 (25.8)	
Hypertension	20 (21.3)	7 (22.6)	
Diabetes	9 (9.6)	3 (9.7)	
Polycystic kidney disease	4 (4.3)	0 (0)	
Others	24 (25.5)	13 (41.9)	
Dialysis modality, n (%)			0.778
Hemodialysis	75 (79.8)	24 (77.4)	
Peritoneal dialysis	19 (20.2)	7 (22.6)	
Duration of dialysis, year, mean (SD)	9.7 (4.4)	10.2 (4.8)	0.585
Preoperative albumin, g/L, mean (SD)	38.6 (2.4)	38.6 (2.8)	0.872
Preoperative ALP, U/L, mean (SD)	486.4 (433.0)	410.0 (332.0)	0.368
Preoperative PTH, pg/mL, median (IQR)	1634.1 (1057.1, 2238.4)	1852.8 (952.1, 2478.3)	0.396
Preoperative calcium, mmol/L, mean (SD)	2.55 (0.21)	2.58 (0.25)	0.490
Preoperative phosphate, mmol/L, mean (SD)	2.31 (0.48)	2.32 (0.58)	0.972
Number of excised parathyroid glands, n (%)			0.775
3	2 (2.1)	2 (6.5)	
4	92 (97.9)	28 (90.3)	
5	0 (0)	1 (3.1)	
Pathological results, n (%)			0.408
Diffuse hyperplasia	53 (56.4)	20 (64.5)	
Nodular hyperplasia	40 (42.6)	11 (35.5)	
Adenoma	1 (1.1)	0 (0)	
Adenocarcinoma	0 (0)	0 (0)	

ALP, alkaline phosphatase; IQR, interquartile range; PTH, parathyroid hormone; SD, standard deviation.

**Table 2:** Preoperative long-term medications for mineral and bone disorder in patients undergoing parathyroidectomy, stratified by the type of postoperative calcium supplementation.

Variable	Group A (prophylactic intravenous calcium supplementation group) (n=94)	Group B (non-prophylactic intravenous calcium supplementation group) (n=31)	P value
Type of calcium-based phosphate binder, n (%)			0.783
Calcium carbonate	26 (27.7)	6 (19.4)	
Calcium acetate	2 (2.1)	2 (6.5)	
Nil	66 (70.2)	23 (74.2)	
Dosage of elemental calcium per day, n (%)			0.655
> 600 mg	8 (8.5)	2 (6.5)	
≤ 600 mg	20 (21.3)	6 (19.4)	
Nil	60 (70.2)	23 (74.2)	
Type of non-calcium-based phosphate binder, n (%)			0.666
Lanthanum	57 (60.6)	21 (67.7)	
Sevelamer	21 (22.3)	5 (16.1)	
Nil	16 (17.0)	5 (16.1)	
Type of vitamin D and analogs, n (%)			0.701
Calcitriol	27 (28.7)	9 (29.0)	
Alfacalcidol	4 (4.3)	1 (3.2)	
Paricalcitol	2 (2.1)	0 (0)	
Nil	61 (64.9)	21 (67.7)	
Dosage of calcitriol per week^*^, n (%)			0.855
> 4 mcg	8 (8.5)	2 (6.5)	
≤ 4 mcg and > 2 mcg	19 (20.2)	6 (19.4)	
≤ 2 mcg	4 (4.3)	2 (6.5)	
Nil	63 (67.0)	21 (67.7)	
Cinacalcet use, n (%)	46 (48.9)	14 (45.2)	0.715
Dosage of cinacalcet per day, n (%)			0.701
75 mg	41 (43.6)	13 (41.9)	
50 mg or 25 mg	5 (5.3)	1 (3.2)	
Nil	48 (51.1)	17 (54.8)	
Duration of cinacalcet use, n (%)			0.647
> 2 years	11 (11.7)	2 (6.5)	
1 year ~ 2 years	27 (28.7)	10 (32.3)	
< 1 year	8 (8.5)	2 (6.5)	
Nil	48 (51.1)	17 (54.8)	

^*^The dosage of alfacalcidol was converted to that of calcitriol at a ratio of 2:1.

**Table 3** present the postoperative laboratory data and outcomes of the two groups. The incidence of postoperative hypocalcemia in group A patients (n=51, 54.3%) was statistically lower than that in group B patients (n=25, 80.6%) (P = 0.009). The serum calcium value of POD1 and POD3 were 2.18 ± 0.39 mmol/L and 2.24 ± 0.48 mmol/L in group A, statistically higher than 1.92 ± 0.36 mmol/L and 1.92 ± 0.39 mmol/L in group B (both P = 0.001). The duration of postoperative hypocalcemia was 4.10 ± 2.36 days in group A and 5.36 ± 2.56 days in group B, with a statistically significant difference (P = 0.037). In patients with hypocalcemia, the time to achieve normal serum calcium levels was 5.14 ± 2.11 days postoperatively in group A versus 6.88 ± 2.70 days postoperatively in group B, with a statistically significant difference (P = 0.003). In group A, 31.9% (n=30) of the cases develop severe hypocalcemia, statistically lower than 61.3% (n=19) in group B (P = 0.004). In addition, patients in group A (n=15, 16.0%) were more prone to hypercalcemia after calcium supplementation compared to group B (n=2, 6.3%), although without statistical difference (P = 0.096). There were no cardiovascular events happened in result of hypercalcemia. For postoperative oral medications, group B received higher doses of calcium carbonate (5.57 ± 3.42 g) and calcitriol (2.61 ± 1.36 mcg) on POD3 compared with group A (3.75 ± 2.88 g and 1.87 ± 1.14 mcg), with statistical differences (P = 0.011 and 0.009). Nevertheless, such differences were not detected in the doses of the two drugs on POD1 or at discharge (all P>0.05). Other complications were rare, and no patients died during the perioperative period. There was no statistical difference between the two groups in persistent SHPT (defined as PTH > 300mmol/L at one week after surgery) and length of hospitalization (both P>0.05).

**Table 3:** Postoperative laboratory data and outcomes of patient undergoing parathyroidectomy, stratified by the type of postoperative calcium supplementation.

Variable	Group A (prophylactic intravenous calcium supplementation group) (n=94)	Group B (non-prophylactic intravenous calcium supplementation group) (n=31)	P value
Postoperative 4 hours PTH, pg/mL, median (IQR)	37.8 (17.4, 73.9)	31.8 (19.3, 80.2)	0.422
Postoperative 4 hours calcium, mmol/L, mean (SD)	2.38 (0.28)	2.37 (0.21)	0.765
Postoperative 4 hours phosphate, mmol/L, mean (SD)	1.99 (0.46)	2.05 (0.43)	0.535
POD1 PTH, pg/mL, median (IQR)	9.2 (4.3, 28.3)	10.5 (5.4, 32.7)	0.258
POD1 calcium, mmol/L, mean (SD)	2.18 (0.39)	1.92 (0.36)	0.001
POD1 phosphate, mmol/L, mean (SD)	1.72 (0.47)	1.69 (0.40)	0.767
POD3 PTH, pg/mL, median (IQR)	3.0 (1.0, 13.4)	7.4 (2.7, 19.5)	0.530
POD3 calcium, mmol/L, mean (SD)	2.24 (0.48)	1.92 (0.39)	0.001
POD3 phosphate, mmol/L, mean (SD)	1.33 (0.35)	1.36 (0.29)	0.698
Postoperative hypocalcemia, n (%)	51 (54.3)	25 (80.6)	0.009
Occurrence time of hypocalcemia, n (%)			0.002
Not happened	43 (45.7)	6 (19.4)	
Within postoperative 24 hours	14 (14.9)	3 (9.7)	
Postoperative 24~48 hours	32 (34.0)	18 (58.1)	
After postoperative 48 hours	5 (5.3)	4 (12.9)	
Duration of hypocalcemia (in hypocalcemia patients), d, mean (SD)	4.10 (2.36)	5.36 (2.56)	0.037
Postoperative days to achieve normal serum calcium levels (in hypocalcemia patients), d, mean (SD)	5.14 (2.11)	6.88 (2.70)	0.003
Severe hypocalcemia^*^, n (%)	30 (31.9)	19 (61.3)	0.004
Hypercalcemia after calcium supplementation, n (%)	15 (16.0)	2 (6.5)	0.096
Postoperative calcium carbonate dosage, g/d, mean (SD)			
POD1	3.78 (1.66)	4.21 (1.12)	0.110
POD3	3.75 (2.88)	5.57 (3.42)	0.011
At discharge	5.94 (3.45)	6.53 (2.73)	0.383
Postoperative calcitriol dosage, mcg/d, mean (SD)			
POD1	1.89 (0.45)	1.94 (0.36)	0.640
POD3	1.87 (1.14)	2.61 (1.36)	0.009
At discharge	1.92 (0.88)	2.16 (0.82)	0.173
Persistent SHPT, n (%)	2 (2.1)	1 (3.2)	1.000
Length of hospitalization, day, mean (SD)	7.36 (2.44)	7.90 (2.53)	0.290

^*^Defined as serum calcium < 1.8 mmol/L.

ALP, alkaline phosphatase; IQR, interquartile range; POD, postoperative day; PTH, parathyroid hormone; SD, standard deviation, SHPT, secondary hyperparathyroidism.

We conducted logistic regression analysis on severe hypocalcemia and hypercalcemia after calcium supplementation aimed to identify their risk factors. For severe hypocalcemia, univariate analysis suggested that age, whether to perform prophylactic intravenous calcium supplementation, and preoperative PTH, calcium and preoperative ALP level were potentially associated with it (P<0.05). After incorporating these variables into multivariate analysis, it was found that non-prophylactic intravenous calcium supplementation(OR 14.360, 95% CI 3.387-60.889, P <0.001), high level of preoperative PTH (OR 1.002, 95% CI 1.000-1.003, P = 0.001) and ALP (OR 1.005, 95% CI 1.002-1.007, P <0.001), and low level of preoperative serum calcium (OR 0.000, 95% CI 0.000-0.021, P <0.001) were risk factors for postoperative severe hypocalcemia (**Table 4**). For hypercalcemia after calcium supplementation, only preoperative high level of ALP (OR 0.996, 95% CI 0.993-0.999, P = 0.018) was found to be a negative predictor in univariate analysis, therefore no multivariate analysis was conducted.

**Table 4:** Analysis of risk factors for severe hypocalcemia after parathyroidectomy using a binary logistic regression analysis.

Variable	Univariate analysis			Multivariate analysis		
	OR	95% CI	P value	OR	95% CI	P value
Age (per 1 year increase)	0.966	0.935-0.999	0.042			
Postoperative prophylactic intravenous calcium supplementation (no vs. yes)	3.378	1.454-7.846	0.005	14.360	3.387-60.889	<0.001
Preoperative PTH (per 1 pg/mL increase)	1.001	1.001-1.002	<0.001	1.002	1.000-1.003	0.001
Preoperative calcium (per 1 mmol/L increase)	0.007	0.001-0.065	<0.001	0.000	0.000-0.021	<0.001
Preoperative ALP (per 1 U/L increase)	1.004	1.003-1.006	<0.001	1.005	1.002-1.007	<0.001

ALP, alkaline phosphatase; CI, confidence index; OR, odds ratio; PTH, parathyroid hormone.

## 4 Discussion

Hypocalcemia is a common complication after parathyroidectomy in SHPT patients, resulting from bone remineralization triggered by rapid decline of PTH (11). The incidence of severe hypocalcemia varied greatly among previous studies for lacking a clear definition (usually ranging from <1.8 mmol/L to <2.1 mmol/L) and differences in perioperative management strategies (12–15). Considering that mild or asymptomatic hypocalcemia is usually harmless (16), we defined severe hypocalcemia as serum calcium <1.8 mmol/L. Currently, the start timing and daily dosage of intravenous calcium supplementation are determined mainly based on the experience of each center because there is no calcium supplementation protocol recognized as a standard of practice. In addition, there were few studies focused on prophylactic intravenous calcium supplementation. In our study, the addition of postoperative prophylactic intravenous calcium, on top of routine oral calcium and calcitriol supplementation, yielded a significant reduction in the incidence of severe hypocalcemia—from 61.3% to 31.9%. Therefore, we propose that immediate postoperative prophylactic intravenous calcium supplementation, in addition to routine oral calcium and calcitriol supplementation, could significantly reduce the incidence of severe hypocalcemia.

Serum calcium commonly reaches its lowest point 2 to 4 days after PTX. The KDIGO guidelines recommended to monitor serum calcium level every 4 to 6 hours within 48–72 hours after surgery, and then twice daily until stable (17). This frequency is impractical to achieve in most centers. Our intravenous calcium supplementation was administered continuously throughout the day, which help avoid rapid fluctuations in serum calcium levels. Therefore, we believe that a monitoring frequency of every 1 to 2 days based on serum calcium levels and hypocalcemia symptoms is acceptable. Furthermore, prophylactic intravenous calcium supplementation reduces the incidence of severe hypocalcemia, thereby contributing to a decreased frequency of serum calcium monitoring and cost savings.

We also noticed that there was huge difference in the calcium requirements among different patients, highlighting the necessity of early identification of high-risk group of severe hypocalcemia. In published studies, elevated preoperative ALP and PTH levels were often reported to be closely associated with postoperative hypocalcemia, while other less common risk factors include longer dialysis duration, advanced age, and hyperkalemia (9, 11, 18, 19). Cheng et al. provided a predictive model combined of preoperative PTH, serum calcium, and ALP levels for the risk of severe hypocalcemia after PTX, and advocated for increased calcium supplementation in high-risk group of patients (20). Serum ALP level partially reflects bone-specific ALP level, the latter of which is considered a better indicator of bone turnover compared with PTH level (21). Consequently, patients with higher serum ALP levels are more likely to exhibit high-turnover bone disease, making them more susceptible to hypocalcemia. Wong et al. proposed an ALP-based calcium supplementation protocol, wherein the intravenous calcium supplement amount on POD1 was determined by preoperative ALP level, and subsequent adjustments were made according to serum calcium level (22). In our study, we also identified high level of preoperative PTH and ALP, and low level of preoperative serum calcium as risk factors for severe hypocalcemia (23, 24). Enhanced calcium supplementation strategies, such as increased initial doses or preoperative supplementation, should be considered for such high-risk patients. Furthermore, a study by Fung et al. demonstrated that compared with immediate surgery, the use of cinacalcet for more than 1 year can significantly reduce preoperative PTH levels, thereby decreasing the incidence of severe hypocalcemia (25). This may represent an alternative strategy for preventing severe hypocalcemia, particularly in individuals reluctant to undergo immediate surgery or those at high risk of developing it.

Intravenous calcium supplementation may be associated with some adverse effects, including hypercalcemia (which can subsequently induce arrhythmias) and phlebitis. The incidence of phlebitis is significantly reduced with the use of CVC. Hypercalcemia is a potential adverse effect of prophylactic intravenous calcium supplementation. Despite continuous monitoring of serum calcium levels, a certain proportion of patients developed hypercalcemia after calcium supplementation in our study. However, we also observed that in most patients whose calcium readings above the normal range, serum calcium levels would gradually decline after pausing intravenous calcium supplementation. Cardiovascular events related to hypercalcemia were also not observed. Therefore, we considered mild hypercalcemia to be safe. Regression analysis revealed that hypercalcemia after calcium supplementation was associated with lower preoperative ALP levels. For these patients, reducing the calcium supplementation doses or replacing with oral calcium agents could be considered. In short, there are differences in calcium requirements among this population. Thus, predicting individual calcium requirements and designing personalized calcium supplementation plans for each patient remain essential for future research.

This study has several limitations. It is a single-center retrospective study, lacking randomization and inherently subject to selection bias and incomplete data, such as the impact of dialysis on serum calcium levels. Furthermore, the absence of long-term follow-up data regarding the recurrence of hypocalcemia and the stability of calcium homeostasis made us unable to analyze long-term hypocalcemia events.

## 5 Conclusions

Our study demonstrates that hypocalcemia is a common complication after PTX. Postoperative prophylactic intravenous calcium supplementation, followed by dynamic dosage adjustments based on serum calcium levels, in addition to routine oral calcium and calcitriol supplementation, could significantly reduce the incidence of severe hypocalcemia without substantially increasing the risk of hypercalcemia. We identified high level of preoperative PTH and ALP, low level of preoperative serum calcium, and non-prophylactic intravenous calcium supplementation as risk factors for severe postoperative hypocalcemia. High level of preoperative ALP is a negative predictor for hypercalcemia after calcium supplementation. Attention should be paid to the management of high-risk group of severe hypocalcemia, which helps improve perioperative safety.

## Data Availability

The original contributions presented in the study are included in the article/**Supplementary Material**, further inquiries can be directed to the corresponding author/s.

## References

[1] Zhang LX Zhang B Liu XY Wang ZM Qi P Zhang TY . Advances in the treatment of secondary and tertiary hyperparathyroidism. Front Endocrinol (Lausanne). (2022) 13:1059828. doi: 10.3389/fendo.2022.1059828, PMID: 36561571 PMC9763452

[2] Brandenburg V Ketteler M . Vitamin D and secondary hyperparathyroidism in chronic kidney disease: A critical appraisal of the past, present, and the future. Nutrients. (2022) 14. doi: 10.3390/nu14153009, PMID: 35893866 PMC9330693

[3] Wesseling-Perry K Salusky IB . Chronic kidney disease: mineral and bone disorder in children. Semin Nephrol. (2013) 33:169–79. doi: 10.1016/j.semnephrol.2012.12.017, PMID: 23465503 PMC4209124

[4] Geng S Kuang Z Peissig PL Page D Maursetter L Hansen KE . Parathyroid hormone independently predicts fracture, vascular events, and death in patients with stage 3 and 4 chronic kidney disease. Osteoporos Int. (2019) 30:2019–25. doi: 10.1007/s00198-019-05033-3, PMID: 31190122

[5] Chou FF Chi SY Wu YJ Chan YC Huang SC . Preoperative work-up and results of parathyroidectomy plus auto-transplantation for the elderly with secondary hyperparathyroidism. Asian J Surg. (2024) 47:880–5. doi: 10.1016/j.asjsur.2023.10.003, PMID: 37989683

[6] Tsai SH Kan WC Jhen RN Chang YM Kao JL Lai HY . Secondary hyperparathyroidism in chronic kidney disease: A narrative review focus on therapeutic strategy. Clin Med (Lond). (2024) 24:100238. doi: 10.1016/j.clinme.2024.100238, PMID: 39208984 PMC11414656

[7] Hiramitsu T Hasegawa Y Futamura K Okada M Goto N Narumi S . Treatment for secondary hyperparathyroidism focusing on parathyroidectomy. Front Endocrinol (Lausanne). (2023) 14:1169793. doi: 10.3389/fendo.2023.1169793, PMID: 37152972 PMC10159274

[8] Steinl GK Kuo JH . Surgical management of secondary hyperparathyroidism. Kidney Int Rep. (2021) 6:254–64. doi: 10.1016/j.ekir.2020.11.023, PMID: 33615051 PMC7879113

[9] Jain N Reilly RF . Hungry bone syndrome. Curr Opin Nephrol Hypertens. (2017) 26:250–5. doi: 10.1097/MNH.0000000000000327, PMID: 28375869

[10] Phimphilai M Inya S Manosroi W . A predictive risk score to diagnose hypocalcemia after parathyroidectomy in patients with secondary hyperparathyroidism: a 22-year retrospective cohort study. Sci Rep. (2022) 12:9548. doi: 10.1038/s41598-022-13880-0, PMID: 35681076 PMC9184730

[11] Carsote M Nistor C . Forestalling hungry bone syndrome after parathyroidectomy in patients with primary and renal hyperparathyroidism. Diagnostics (Basel). (2023) 13. doi: 10.3390/diagnostics13111953, PMID: 37296804 PMC10252569

[12] Amjad W Ginzberg SP Passman JE Heintz J Kelz RR Wachtel H . Predictive risk score for postparathyroidectomy hungry bone syndrome in patients with secondary hyperparathyroidism. J Clin Endocrinol Metab. (2024) 109:603–10. doi: 10.1210/clinem/dgad636, PMID: 37897423

[13] Ding C Guo Y Mo Q Ma J . Prediction model of postoperative severe hypocalcemia in patients with secondary hyperparathyroidism based on logistic regression and XGBoost algorithm. Comput Math Methods Med. (2022) 2022:8752826. doi: 10.1155/2022/8752826, PMID: 35924110 PMC9343187

[14] He C Zhang Y Li L Cheng G Zhang W Tang Y . Risk Factor Analysis and Prediction of Severe Hypocalcemia after Total Parathyroidectomy without Auto-Transplantation in Patients with Secondary Hyperparathyroidism. Int J Endocrinol. (2023) 2023:1901697. doi: 10.1155/2023/1901697, PMID: 36700169 PMC9870689

[15] Zou Y Zhang N Tang Y Zhan Z Yang M Lu Y . Predictive markers for severe hypocalcemia in dialysis patients with secondary hyperparathyroidism after near-total parathyroidectomy. Ann Palliat Med. (2021) 10:10712–9. doi: 10.21037/apm-21-2509, PMID: 34763432

[16] Lau WL Obi Y Kalantar-Zadeh K . Parathyroidectomy in the management of secondary hyperparathyroidism. Clin J Am Soc Nephrology: CJASN. (2018) 13:952–61. doi: 10.2215/cjn.10390917, PMID: 29523679 PMC5989682

[17] Kidney Disease: Improving Global Outcomes CKDMBDUWG . KDIGO 2017 clinical practice guideline update for the diagnosis, evaluation, prevention, and treatment of chronic kidney disease-mineral and bone disorder (CKD-MBD). Kidney Int Suppl. (2017) 7:1–59. doi: 10.1016/j.kisu.2017.04.001, PMID: 30675420 PMC6340919

[18] Bi T Bai SJ Cheng GM Feng XD Zhang W . Predictive analysis of severe hypocalcemia following total parathyroidectomy for renal secondary hyperparathyroidism. Eur Rev Med Pharmacol Sci. (2024) 28:2217–23. doi: 10.26355/eurrev_202403_35726, PMID: 38567585

[19] Tan ZK Looi WL Chen F Yeo SC Bairy M . Determinants of severe hypocalcemia after parathyroidectomy in patients with end-stage kidney disease and renal hyperparathyroidism: A retrospective cohort study. J Clin Med. (2025) 14. doi: 10.3390/jcm14020379, PMID: 39860385 PMC11765800

[20] Cheng J Lv Y Zhang L Liu Y . Construction and validation of a predictive model for hypocalcemia after parathyroidectomy in patients with secondary hyperparathyroidism. Front Endocrinol (Lausanne). (2022) 13:1040264. doi: 10.3389/fendo.2022.1040264, PMID: 36531501 PMC9748676

[21] Haarhaus M Cianciolo G Barbuto S La Manna G Gasperoni L Tripepi G . Alkaline phosphatase: an old friend as treatment target for cardiovascular and mineral bone disorders in chronic kidney disease. Nutrients. (2022) 14. doi: 10.3390/nu14102124, PMID: 35631265 PMC9144546

[22] Wong J Fu WH Lim ELA Ng CFJ Choong HL . Hungry bone syndrome after parathyroidectomy in end-stage renal disease patients: review of an alkaline phosphatase-based treatment protocol. Int Urol Nephrol. (2020) 52:557–64. doi: 10.1007/s11255-020-02387-0, PMID: 32016909

[23] Wen P Xu L Zhao S Gan W Hou D Zhang L . Risk factors for severe hypocalcemia in patients with secondary hyperparathyroidism after total parathyroidectomy. Int J Endocrinol. (2021) 2021:6613659. doi: 10.1155/2021/6613659, PMID: 33868402 PMC8035008

[24] Yeh H Yeh H Chiang CC Yen JC Wang IK Liu SH . Hungry bone syndrome in peritoneal dialysis patients after parathyroid surgery. Endocr Connect. (2023) 12. doi: 10.1530/EC-23-0107, PMID: 37606078 PMC10563628

[25] Fung MMH Tam DS Lui DTW Lang BHH . Pre-operative cinacalcet administration reduces immediate post-operative hypocalcemia following total parathyroidectomy in severe renal hyperparathyroidism. World J Surgery. (2023) 47:1986–94. doi: 10.1007/s00268-023-07030-4, PMID: 37140608

